# Drenched Pages: A Primer on Wet Books

**DOI:** 10.3390/biology14080911

**Published:** 2025-07-22

**Authors:** Islam El Jaddaoui, Kayo Denda, Hassan Ghazal, Joan W. Bennett

**Affiliations:** 1 Laboratory of Human Pathologies Biology, Department of Biology, Faculty of Sciences, University Mohammed V, Rabat 10000, Morocco; 2 Genomic Center of Human Pathologies, Faculty of Medicine and Pharmacy, University Mohammed V, Rabat 10000, Morocco; 3 Department of Biochemistry and Microbiology, Rutgers, The State University of New Jersey, New Brunswick, NJ 08901, USA; 4 New Brunswick Libraries, Rutgers, The State University of New Jersey, New Brunswick, NJ 08901, USA; 5 Laboratory of Precision Medicine & One Health (MedPreOne), School of Medicine, Mohammed VI University of Sciences & Health, Casablanca 82403, Morocco; 6 Laboratory of Sports Sciences and Performance Optimization, Department of Sports Sciences, Royal Institute of Executive Management, Salé 10102, Morocco; 7 National Center for Scientific and Technical Research, Rabat 10102, Morocco

**Keywords:** libraries, fungi, mold, mycology, preservation, biodeterioration, human health

## Abstract

Molds readily grow on wet books, documents, and other library materials where they ruin them chemically, mechanically, and aesthetically. Poor maintenance of libraries, failures of Heating, Ventilation, and Air Conditioning (HVAC) systems, roof leaks, and storm damage leading to flooding can all result in accelerated fungal growth. Moreover, when fungal spores are present at high concentrations in the air, they can be linked to severe respiratory conditions and possibly to other adverse health effects in humans. Climate change and the accompanying storms and floods are making the dual potential of fungi to biodegrade library holdings and harm human health more common. This essay is intended for microbiologists without much background in mycology who are called in to help librarians who are dealing with mold outbreaks in libraries. Our goal is to demystify aspects of fungal taxonomy, morphology, and nomenclature while also recommending guidelines for minimizing mold contamination in library collections.

## Simple Summary

Mold quietly invades damp books and historical documents, leaving behind stains, odors, and structural decay. From leaky roofs to storm-driven floods, environmental factors can turn libraries and archives into ideal breeding grounds for these destructive fungi. Beyond the damage to irreplaceable cultural treasures, mold spores released into the air can pose serious health risks. This article focuses on the hidden world of mold in libraries and provides accessible guidance for scientists seeking to understand and reduce mold contamination in cultural collections, with the goal of protecting both heritage materials and public health.

## 1. Introduction

For those who find libraries and their contents indispensable, the work by Lucien Polastron, titled *Books on Fire: The Destruction of Libraries Throughout History* (2004) [1], is a depressing read. Page after page of this text documents the fiery destruction of libraries in Alexandria, Athens, Cordoba, Bagdad, Rome, Constantinople, and other seats of ancient learning and then moves through the ensuing millennia, chronicling the obliteration of priceless scholarship by conquerors, thieves, religious fanatics, and other book burners. He writes that “fire…was responsible for most of the library destructions in history” [1]. Then, several hundred pages later, he briefly contradicts himself and writes, “Water, however, damages books more surely than fire…”. This is easily overlooked because “water seems weak…” and then goes on to explain: “The phrase ‘books on fire’ obviously piques the imagination more than ‘drenched pages’” [1]. The only other mention of water damage in this comprehensive book comes where reference to the “ferocious humidity” in the city of Alexandria is followed by speculation that the famous lost library there was similar to those in Pergamum and Ephesus, where “braziers sent currents of hot air through baked clay pipes to offset the moldiness” [1]. Curiously, despite these brief acknowledgments of the destructive power of water and possible fungal contamination, the index of the book does not have a single entry for “mold”, even while it references “Molotov cocktails”, “monastic libraries”, and “Mongols” [1].

## 2. Libraries, Librarians, and Mold

The professional literature describing the role of librarians emphasizes education, outreach, access, and literacy. In reality, many head librarians find themselves in far less lofty roles—roles that they did not expect to fulfill and for which they are not necessarily prepared—such as de facto directors of physical facilities who are responsible for routine maintenance and reactive repairs. In particular, librarians must be vigilant about detecting unusual levels of dampness that can be caused by something as simple as condensation or due to “unforeseen” problems with leaking pipes, open windows, storm damage, and natural disasters. Water can harm almost all library resources in all formats, from print to digital. Every component of a book, including its sizing, glue, and binding can support fungal growth, causing subsequent biodeterioration. Exposure to high levels of indoor fungal proliferation may also threaten human health [2,3,4,5]. This paper is intended as a primer for microbiologists who have little or no training in mycology, but who nevertheless are called in as professionals to assess water and mold damage in libraries. In the first section of this paper, we present a brief introduction to mycology with emphasis on the jargon that most bacteriologists, virologists, and protozoologists have never learned or have forgotten. Next, we present an overview of the study of mold spores and other biological particulates in the air, followed by a section on how to remediate water-damaged materials. We conclude with a section on the association between indoor molds and possible threats to human health.

## 3. Basic Concepts of Mycology

Mycology is the study of fungi. The best-known fungi are macroscopic (e.g., mushrooms, toadstools, shelf fungi, and truffles), and traditional mycologists developed taxonomic and other disciplinary traditions apart from microbiology. Nevertheless, there are far more microscopic fungi, such as single-celled yeasts, and the more common filamentous species colloquially called molds and mildews, than there are macroscopic fungi [6]. Unfortunately, their study is often poorly represented in microbiology curricula.

Fungal taxonomy follows a hierarchical system of classification, including ranks such as kingdom, phylum, class, order, family, genus, and species. It is important to distinguish between formal taxonomic categories and morphological or ecological groupings such as “molds” or “yeasts.” These latter terms refer to growth forms or lifestyles rather than specific taxonomic ranks. The primary objectives of fungal taxonomy are to identify and describe all fungal species, organize them based on their evolutionary relationships, and develop tools for accurate identification [7]. At present, fungi are classified into five recognized divisions. Chytridiomycota includes various fungi adapted to aquatic environments. Glomeromycota form symbiotic associations with plant roots and generally have limited relevance to material biodeterioration. Zygomycota contains several fungi known for spoiling grains, fruits, and vegetables and can occasionally colonize museum objects opportunistically. Basidiomycota, which includes mushrooms and toadstools, is notable for containing *Serpula lacrymans*, a major agent of wood decay in historical buildings and churches. However, the majority of molds involved in the deterioration of cultural heritage materials are part of the Ascomycota division [8].

Fungi develop both vegetative and reproductive structures, each with distinct morphological features used for identification (Table 1). Diagnostic characteristics include the shape, size, pigmentation, and septation of conidia or spores; the architecture and branching of conidiophores; and the organization of reproductive elements like phialides [9]. The main vegetative component, the hypha, is a threadlike filament that may be unicellular or multicellular, typically ranging from 1.5 to 12 μm in diameter. A network of hyphae is called a mycelium. Hyphae can be continuous tubes or divided into cells by septa—walls that generally have openings allowing cytoplasmic flow between cells. Fungal spores, the reproductive units, can vary widely in color, shape, and cellular complexity, depending on the species and conditions, and generally measure between 5 and 20 μm. Spores may form by splitting hyphal walls, internally within hyphal cells, or by budding from hyphal protrusions. In more complex forms, spores are produced on specialized erect branches called conidiophores, where they appear singly or in structures like chains or heads [10].

Aside from a few macroscopic features that can be seen with the naked eye, microscopy is essential for examining fungal morphology and cellular structures. Different types of microscopes reveal various aspects of fungal anatomy. Techniques commonly used in morphological studies include traditional light microscopy, electron microscopy, fluorescence microscopy, phase contrast microscopy, confocal laser scanning microscopy, and atomic force microscopy. Among these, compound light microscopy remains the most widely used method for observing fungal structures [11].

For non-specialists involved in identifying fungi in library materials, several authoritative resources provide essential guidance on fungal morphology and taxonomy. Classic works such as *Illustrated Genera of Imperfect Fungi* [12], *Dematiaceous Hyphomycetes* [13], and *The Identification of Fungi: An Illustrated Introduction With Keys, Glossary, And Guide to Literature* [14] offer accessible keys, illustrations, and terminology useful for characterizing common fungal contaminants. In addition, *Compendium of Soil Fungi* [15] remains a fundamental reference for environmental and indoor fungi frequently encountered in biodeterioration contexts. Complementing these texts, online platforms such as the University of Adelaide’s Mycology Online (https://www.adelaide.edu.au/mycology/ (accessed on 5 July 2025)) and the MycoBank (https://www.mycobank.org/ (accessed on 5 July 2025)) database offer up-to-date, searchable tools and visual references that are particularly useful for non-experts seeking reliable taxonomic and ecological information.

Morphology-based taxonomy can sometimes fail to accurately distinguish species due to overlapping physical traits. Additionally, sampling errors and the subjective experience of taxonomists may influence morphological identification. Some fungal taxa do not grow well or fail to produce reproductive structures on artificial media and may rarely form sexual or asexual structures in natural environments. As a result, such taxa can be missed by traditional morphology-based methods despite their potential significance within fungal communities [11]. These limitations can be overcome through molecular analyses. Molecular techniques, especially polymerase chain reaction (PCR) technology, have transformed fungal molecular biology and diagnostics. Integrating molecular methods with conventional morphology-based taxonomy enhances the ability to differentiate fungal species and varieties. Efforts are underway to develop genomic and genetic marker databases for molecular barcoding, enabling a more comprehensive exploration of fungal diversity using bioinformatics tools [16].

Neither mold nor mildew has a precise scientific definition. Currently, mildew usually refers to fungal growth that looks white, powdery, woolly, fuzzy, and/or cottony, while mold is used more generically to refer to all flat microscopic fungal growth, encompassing fungi with spores of many colors and forms. This paper follows contemporary language practices and uses mold generically to refer to almost all contamination of library materials by microscopic filamentous fungi. However, in the past, mildew was often encountered in the published literature about fungal contamination of books [17,18], and is still common in vernacular speech and in advertisements for commercial products aimed at cleaning up fungal contamination. The term mildew is also used with respect to many plant pathogens in the family Peronosporaceae, a group of fungal-like “water molds” that parasitize plants [19].

Many different species of molds thrive on paper, book bindings, cardboard, wood, ceiling tiles, wallpaper, fabric, carpets, upholstery, drywalls, paints, and insulation [20,21]. When viewed under the microscope, molds are composed of *hyphae* (sing = *hypha*). These filamentous cells mechanically penetrate materials while simultaneously excreting acids and enzymes that degrade their substrates. For easily readable general introductions to mycology, we recommend *Magical Mushrooms, Mischievous Molds* by George Hudler (1998) [22]; *Mycophilia* by Eugenia Bone (2011) [23]; and the gorgeously written *Entangled Life: How Fungi Make Our World, Change Our Minds Shape Our Futures* by Merlin Sheldrake (2020) [24]. Useful references for background information on fungi with emphasis on the identification of molds and/or their relevance to library holdings are found in the Reference section denoted by the symbol *.

**Table 1:** Main fungal genera isolated from libraries and their key morphological characteristics.

Fungal Genera	Notable Features	References
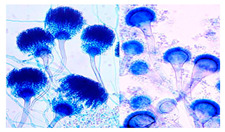 *Aspergillus* sp. ^a^	*Aspergillus* is characterized by the production of conidiophores, which arise from foot cells of the hyphae. These conidiophores terminate in a swollen vesicle that bears one or two layers of phialides. The phialides produce chains of spores, which are typically globose to subglobose, unicellular, and often rough-walled. Colony morphology varies by species but generally shows rapid growth and pigmentation ranging from green, yellow, brown, to black.	[25,26]
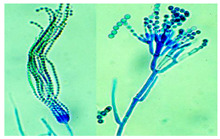 *Penicillium* sp. ^a^	*Penicillium* species are characterized by their fast-growing, filamentous colonies with a typically velvety to powdery texture and colors ranging from blue-green to grey or white. Microscopically, they produce brush-like conidiophores (penicilli) composed of a stipe, a whorl of metulae, and chains of phialides that give rise to dry, globose to elliptical conidia in basipetal succession. The conidiophores may be monoverticillate, biverticillate, or terverticillate depending on the species. The conidia are usually smooth to finely roughened and pigmented.	[27]
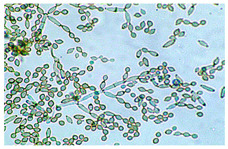 *Cladosporium* sp. ^a^	*Cladosporium* species are characterized by their darkly pigmented hyphae, conidiophores, and conidia. Conidiophores are erect, often bent, and may be covered with nodules, with branching typically occurring apically. They produce conidia in branched, acropetal chains. The conidia are variable in shape, ranging from ellipsoidal to cylindrical or ovoid, and may be smooth, rough, or finely ornamented. A distinctive diagnostic feature of *Cladosporium* is a thickened, darkened scar at the point of conidial attachment, with a central dome.	[28,29]
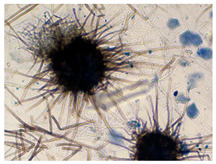 *Chaetomium* sp. ^a^	*Chaetomium* species have dark perithecia that are typically globose to ovate, with an opening, and covered with distinctive terminal hairs, which may be straight, coiled, or branched. The asci are single-walled, evanescent, club or spindle shaped, and usually contain eight ascospores. Ascospores are brown, lemon-shaped, bilaterally flattened, and commonly possess one or two germ pores.	[30,31]
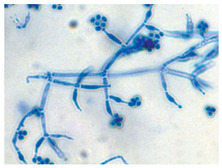 *Trichoderma* sp. ^a^	*Trichoderma* is characterized by rapidly growing, often green colonies with compact or tufted mycelium. The conidiophores are typically highly branched, forming pyramidal structures, and bear phialides that are flask-shaped, occurring singly or in whorls. These phialides produce green, unicellular conidia that are subglobose to ellipsoidal, smooth to finely roughened, and accumulate in slimy heads.	[32,33]
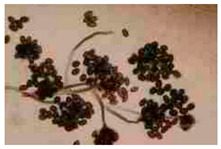 *Stachybotrys* sp. ^b^	*Stachybotrys* is characterized by grape-like bunches of septate conidiophores that are often branched at the base and bear terminal or intercalary phialides. The phialides are typically swollen at the base and taper to a narrow neck, producing ellipsoidal to obovoid, smooth- to rough-walled conidia that form in slimy, dark green to dark brown or black heads.	[34,35]
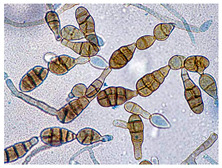 *Alternaria* sp. ^a^	*Alternaria* species are characterized by the production of dark brown to olive conidia that are typically multicellular with both transverse and longitudinal septa. The conidia are usually obclavate to ellipsoid in shape and often possess a tapered beak at the apex. These spores are formed in chains by acropetal development on branched conidiophores that arise from septate, hyaline to pale green hyphae. The conidial surface may be smooth or ornamented, and conidia are typically produced in characteristic chains or clusters. Morphological variation, including septation patterns and beak length, is important for species delimitation within the genus.	[36,37]

^a^ Images sourced from: https://www.adelaide.edu.au/mycology/ (accessed on 5 July 2025); ^b^ image sourced from: https://www.engineeringenvironmental.com/environmental-services/different-mold-types.html (accessed on 5 July 2025); sp. after the genus name indicates that the fungal genus is known, but the species was not identified.

### 3.1. Aeromycology

Aeromycology is a sub-disciplinary field that focuses on fungal elements that are specifically found in indoor and outdoor air [38]. The more general discipline, aerobiology, sometimes called atmospheric biology, is the study of all airborne biological particles, including bacteria, bird and rodent droppings, fungal spores and cell fragments, insect body parts, mites, plant pollen, and other plant particles [39]. In outdoor environments, the abundance of fungal particulates varies with climate, weather, humidity, temperature, wind, vegetation type, and even time of day. In indoor environments such as libraries, spore concentrations vary with construction materials, the age of the buildings, cleaning practices, the number and movement of people, ventilation associated with heating and cooling systems, and the presence of pests, plants, and food. Moisture is the single most important factor affecting indoor fungal growth and spore concentrations [38,40,41,42,43]. Much of the classical work in aeromycology focuses on the allergenic potential of airborne fungal spores associated with inflammation and allergy [44]. Exposure to indoor molds is also believed to contribute to an elusive and somewhat controversial condition called “Sick Building Syndrome” [3,45]. The *Manual of Environmental Microbiology* has several excellent chapters on aerobiology [46]; the American Society of Heating, Refrigerating, and Air-Conditioning Engineers (ASHRAE), the American Industrial Hygiene Association, and several professional societies also produce valuable resources on indoor air quality [47,48,49]. If there is a silver lining to the COVID-19 pandemic, it is that it has made the general public aware of the need for appropriate ventilation and filtration in controlling the quality of indoor air [50,51]. Attention to ventilation to maintain air quality is mentioned in the book *Designing the Libraries for the 21st Century*. The book emphasizes the increased focus on air quality in libraries and the importance of operable windows to allow fresh air into the buildings. This is not only desirable for the librarians and library staff working in the buildings and users studying in library space, but also for the health of the collection, preventing conditions for mold growth [52].

### 3.2. Understanding Fungal Nomenclature

In mycology, as in other parts of biology, each binomial name serves as a useful verbal shorthand to access a great deal of functional knowledge. For those who want to learn more about the vagaries of biological nomenclature, we recommend the accessible books *The Naming of the Shrew: A Curious History of Latin Names* [53] and *The Art of Naming* [54].

Of the more than 100,000 species of named fungi, only about 150 are commonly identified from indoor environments, including libraries [55]. Commonly encountered genera isolated from indoor environments include *Alternaria*, *Aspergillus*, *Aureobasidium*, *Cladosporium*, and *Penicillium* [56]. It is often difficult to distinguish species within a genus, and there are several hundred species each of *Aspergillus* and *Penicillium* [26,57,58]. Most of these species reproduce largely by asexual spores, and in formal fungal taxonomy, this lifestyle is given a variety of specialized names: *Fungi Imperfecti*, *Hyphomycetes*, or *Deuteromycetes* (“second fungi”). Other labels without formal taxonomic rank include “anamorphic fungi” and “mitosporic fungi” [9,59,60,61,62]. Even mycologists are often confused by the proliferation of this specialist language. Table 2 provides a glossary defining terms commonly used in the mycological literature relevant to indoor mold contamination.

In the past, fungal species have undergone many confusing nomenclatural changes, and they continue to do so today. Molecular technologies have revolutionized species identification, revealing new relationships. Until 2013, the rules of botanical nomenclature under which fungi were governed allowed different genus names for sexual and asexual forms of the same species. Nevertheless, because of the ongoing debates around classification and nomenclature, many people who studied fungi deliberately ignored the formal rules of fungal taxonomy and practiced what Hibbett and Taylor (2013) [63] called “nomenclatural disobedience” and gave the same genus name to both asexual and sexual species. Although the confusing dual nomenclature for fungi has been abandoned, the mycological community continues to debate the best way to name and classify fungi that reproduce asexually [63]. The ongoing disputes have created considerable instability in nomenclatural conventions. A good example of the confusion caused by arcane nomenclature comes from the study of molds isolated from a water-damaged Venetian library [64]. The principal species isolated from this library was identified as *Eurotium halophilicum*. *Eurotium* is the “sexual” (“teleomorph”) name for the fungus also known as *Aspergillus*. Obviously, it is confusing for non-mycologists to meet the well-known genus *Aspergillus* under a different name [65], and these taxonomic fine points make it difficult to access the specialized knowledge of mycologists.

In many indoor environments, including libraries, the single most common genera isolated from the air are *Alternaria*, *Aspergillus*, *Chaetomium*, *Cladosporium*, *Colletotrichum*, *Fusarium*, *Penicillium*, and *Trichoderma*, listed in alphabetic order [66,67]. *Cladosporium* and *Alternaria* grow mainly outdoors, while *Aspergillus* and *Penicillium* are more commonly found indoors. Some experts consider *Aspergillus* to be the single most important genus of indoor molds [68], and in a review of 54 published papers on fungi isolated from libraries, it was the single most reported genus [67].

**Table 2:** Glossary of terms encountered in mycology.

Term	Definition
Air sampler	A device used to collect a predetermined volume of air [69]. It mainly consists of an inlet directing air into a collector, a filter to winnow out larger particles, a collector where the sample is deposited, typically an agar-based growth medium, a flowmeter to calibrate the airflow, and a pump to suck the air into the apparatus [70].
Allergy	A chronic condition involving an abnormal immunological reaction to an ordinarily harmless substance called an allergen. A hypersensitivity reaction in response to otherwise inert substances present in the environment [71].
Anamorph	The anamorph is the asexual state in the fungal life cycle [72].
Apothecium	An open, cup-shaped fruiting body found in certain fungi, which bears asci (spore-producing cells) exposed on the upper surface [72].
Ascus	pl. Asci, a sac-like structure where sexual spores (ascospores) are formed in Ascomycota fungi [73].
Aspergillosis	*Refers to a group of diseases caused by fungi of the genus* Aspergillus, *ranging from localized infections to potentially lethal systemic diseases affecting the lung and other organs [74]*
Asthma	Difficulty with breathing; *spec.* a disease of respiration; a chronic lung disease that affects the lower respiratory tract, caused by inflammation and narrowing of the airways characterized by intermittent paroxysms of difficult breathing, with a wheezing sound, a sense of constriction in the chest, cough, and expectoration [75,76].
Ascomycetes	A class within the fungal kingdom characterized by hyphae with cross walls (septate hyphae) and sexual spores (ascospores) produced within a structure called an ascus on a fruiting body called ascomata. This group of fungi has the greatest number of currently recognized fungal species. When classified as a phylum this group is called the Ascomycota [72].
Atopy	A genetic tendency to initiate an immune response against various allergens and antigens leading to overproduction of immunoglobulin E, usually associated with an increased hypersensitivity response to common inhaled or ingested allergens [77].
Basidiomycetes	A class within the fungal kingdom characterized by hyphae with cross walls (septate hyphae) and sexual spores (basidiospores) produced within a specialized structure called a basidium. When classified as a phylum, this group is called the Basidiomycota. Basidiomycetes include the majority of macroscopic fungi such as mushrooms and toadstools. When basidiospores are found inside a building, they are often an indication of decayed wood [78,79].
Clavate	Club-shaped; thicker at the apex than at the base [61].
Cleistothecium	A closed, spherical fruiting body found in some Ascomycota fungi, in which the asci (spore-producing structures) are completely enclosed [72].
Conidiophore	A specialized fungal hypha that bears conidiospores [80].
Conidium	Plural conidia, the most common form of asexual spore [81] found in fungi with septate hyphae.
CFU	Colony-forming unit (CFU) is a measure of viable cells in a particular sample in which a colony represents a macroscopic group of cells (the “colony”) derived from a single progenitor cell [82]. Because of the hyphal nature of filamentous fungi, CFUs are less reliable indices of cell number than they are for bacteria or yeasts.
Dematiaceous fungi	Heterogenous group of environmental molds characterized by dark pigmentation [83].
Eumycota	Also called “true fungi”, this is a taxonomic group that includes all organisms currently recognized as fungi [84].
Fusiform	Spindle shaped [61].
Foxing	The term “foxing” generally refers to small, roundish spot stains of reddish or yellowish brown color, found in paper or other fiber-base materials [85].
Geniculate	Bent like a knee [61].
Genus	Plural genera, a main category of biological classification ranking below family and above species. It contains related species. Examples of genus names include Aspergillus, Cladosporium, and Penicillium. The genus name is the first of the double names used worldwide to designate a given organism [86].
Germination	A process that activates the spore, transitioning it from a dormant state to an actively growing organism capable of undergoing sexual or asexual reproduction [87].
Hilum	Refers to a scar or indentation on a fungal spore, specifically at the point where it was attached to its parent structure [61].
Hyphae	Fungal cells consisting of long slender branched filaments, usually having transverse cross walls (septa). The ends of the hyphae extend to form an expanding network of mycelium [88,89].
Hyphomycetes	An artificial group of filamentous fungi that reproduce asexually by producing spores on hyphae or hyphal aggregations. These fungi lack a known sexual stage in their life cycle and reproduce only through mitotic spore formation. They are collectively referred to as “Deuteromycetes,” “Fungi Imperfecti,” or “Imperfect fungi” due to the absence of observed sexual reproduction [60,90].
Hypersensitivity pneumonitis	Hypersensitivity pneumonitis is a complex interstitial lung disease characterized by an exaggerated immunological reaction of the lung parenchyma in response to the inhalation of an allergen [91].
Meiospores	Spores formed through meiosis, typically involved in the sexual reproduction of fungi, resulting in genetically diverse haploid cells (i.e., Ascospores, Basidiospores, Zygospores) [92].
Mitospores	Asexual spores produced by mitosis, allowing fungi to reproduce rapidly without genetic recombination [93].
Mycelium	The vegetative tissue (thallus) of a fungus, consisting of a network of fine filaments (hyphae); the mass of hyphae that form the vegetative part of a fungus [94].
Mycotoxins	A highly diverse group of secondary metabolites produced by microscopic fungi that are toxic to humans and other animals [95].
Mycotoxicosis	Disease caused by mycotoxins, usually due to the ingestion of a fungal mycotoxin, but also due to inhalation and skin exposure [95,96].
Nodulose	Adjective describing something that has or is covered in small, rounded bumps or lumps, similar to nodules. It essentially means “having minute nodules” [61].
Ostiole	Small hole or opening through which algae or fungi release their mature spores [61].
Phialides	Specialized cells that produce conidia [97].
Relative humidity	The moisture present in the air, expressed as a percentage of the amount of moisture held in the atmosphere at a given pressure and temperature without condensation [98].
Taxonomy	A scientific discipline that provides a universal naming and classification system where items are arranged according to a hierarchical framework [99,100]. The rules of fungal nomenclature are currently governed by the International Code of Nomenclature for Algae, Fungi, and Plants (Shenzhen Code) [101].
Teleomorph	Sexual state in the fungal life cycle, also referred to as the “perfect state” [102].
Verrucose	Means covered with or resembling warts. It describes a surface that is rough and bumpy due to wart-like projections or outgrowths [61].
Water activity (aw)	Refers to the amount of water available in a substrate for biological or chemical processes. It is expressed as the ratio between the vapor pressure of water in the material and the vapor pressure of pure water at the same temperature, ranging from 0.0 aw (completely dry) to 1.0 aw (pure water) [103].
Xerophile	An organism adapted for life with a limited supply of water. Xerophiles can grow and multiply at low water activity (aw) levels. Many species of fungi found in libraries are xerophiles [104,105,106].
Yeast	Single-celled, round or oval fungus that reproduces by budding or fission [107].

## 4. Spores, Germination, and Reproduction of Fungi

Fungi usually reproduce and disperse themselves via spores. Spores are to fungi what seeds are to plants, and like seeds, spores can remain dormant for years, germinating only when they encounter appropriate moisture conditions. When biologists study the particles found in the air, fungal spores and other fungal propagules often form the major component of bioaerosols. This is because during evolution fungi have exploited the wind for dispersal more thoroughly than any other group of organisms [6,108]. Globally, it has been estimated that fungi “produce around fifty megatons of spores each year–equivalent to the weight of 500,000 blue whales–making them the largest source of living particles in the air” [24]. Spores that settle in carpeting, ventilation ducts, and other indoor reservoirs can become sources of bioaerosols. The ubiquitous presence of fungal propagules in the atmosphere means that when sufficient water and a suitable substrate become available for spore germination, molds, yeasts, and other fungi will rapidly germinate, grow, and form colonies.

As a group, fungi have three attributes that make them especially suited for growth in human-built environments: (1) they form astronomical numbers of spores that travel through the atmosphere and easily penetrate buildings, thereby providing inocula for future colony formation; (2) they produce numerous extracellular enzymes that are among the major agents of biodegradation on planet earth; and (3) many fungi are capable of growing at extremely low levels of water, i.e., they are “xerophilic”. Further, fungi have a unique form of nutrition in which they excrete both acids and extracellular enzymes into the environment. After the acids and enzymes have broken down complex organic compounds, the degradation products are reabsorbed as food for growth. This mode of nutrition is technically called “osmotrophic” and is popularly described as fungi “having their stomachs on the outside” [109] Different fungal species have evolved to produce different suites of extracellular enzymes that break down a wide range of substrates, making them the greatest recyclers of nature. Fungi are efficient decomposers and there is usually at least one fungus capable of degrading any type of natural material. Fungi play a vital ecological role in the breakdown of carbon and nitrogen molecules into components that can be used by themselves and other species. Moreover, fungi are exceptionally adept at breaking down cellulose and lignin, which are key components of wood and paper [81].

Across the whole fungal kingdom, fungi have evolved species that can utilize a huge number of different substrates, although individual species tend to be selective in their nutritional requirements [110,111]. The same metabolic diversity that enables members of the fungal kingdom to serve as important global recyclers in nature is problematic in human-built environments, where fungi become the major agents of biodeterioration. Many thrive on paper, cloth, glues, sizing, leather, and parchment. Once molds grow on a book or other document, they change the chemical composition of their substrates and weaken the material. In addition, many fungi secrete colored pigments that cause permanent stains [112,113].

Foxing describes small, typically round spots with reddish or yellowish-brown discoloration that appear on paper and similar fibrous materials. Because there is no universally accepted definition, it is sometimes unclear whether certain localized stains qualify as foxing [85]. The origin of foxing has been a topic of debate. Although abiotic factors—such as moisture, metal contamination, and paper acidity—are known to influence its development, growing evidence points to a biological cause as well [114]. Fungal activity, in particular, has been linked to foxing, with stains resulting in various hues. These colors may derive from fungal structures, their metabolic by-products, or chemical interactions between those metabolites and the paper substrate [85].

The ability of fungi to secrete degradative enzymes and acids that are essential for major ecological cycles in nature leads to food spoilage, wood rot, the decomposition of paper and fabrics, and the destruction of cultural heritage artifacts. Species that can grow on wood can degrade wooden building materials and render buildings unsafe [115,116]. Fungi are particularly good at degrading cellulose, the single most abundant plant-derived substance on earth [117,118]. Paper is largely composed of cellulose. The main fungal genera (e.g., *Aspergillus*, *Penicillium*, *Trichoderma*) that contaminate libraries are also used in biotechnology because they can be harnessed for the industrial production of cellulose-degrading enzymes (cellulases) [119,120].

Many fungi can grow at much lower levels of water availability than other organisms. Depending on how little water these species require, they are described as either xerotolerant (“dry tolerating”) or xerophilic (“dry loving”) [8]. Xerophilic molds thrive at extremely low water activity and are the most frequently identified species associated with libraries, meaning that only mild dampening of materials may be sufficient for fungal growth. The main exception to this generalization is *Stachybotrys chartarum*, a slow-growing, toxin-producing, black-spored species that requires relatively high moisture levels to grow. For this reason, the identification of *S. chartarum* in indoor environments is an indication of severe water damage [121].

The damage caused by fungal degradation of library materials depends on both the fungal species involved and the nature of the substrate. Although many studies have successfully identified fungi associated with these changes, uncertainty often remains as to whether the detected species is the primary agent responsible for the damage [66]. Fungi exhibit varying levels of cellulolytic activity depending on their species and strains. In addition to cellulases, many fungi produce other extracellular enzymes such as amylases and proteases, which break down paper components and binding agents. They also secrete colored metabolites in a range of hues, which can cause discoloration and damage to documents and artworks [66]. These species can produce strong odors (*Trichoderma* spp.), colored stains (*Chaetomium* spp. and *Epicoccum* spp.), or toxic compounds (*Stachybotrys* spp.) [8]. Given their potential to cause biodeterioration, accurate species identification and detailed characterization of their damaging capabilities are essential for developing effective preservation strategies for cultural heritage materials worldwide [122]. Nevertheless, identifying the specific microorganisms responsible for paper deterioration is complex and has often resulted in confusion and extensive lists of potential culprits [123].

Fungal infestations are often associated with the presence of insects and mites. Many of these arthropods (e.g., silverfish and “book lice”) feed on fungal spores or book and document components (e.g., beetles, cockroaches, and termites) and can spread fungal infestations in libraries. The damage caused by arthropods to library collection is beyond the purview of this review. For specific information on insect and mite damage, see Florian [124] and Querner [125].

## 5. Disaster Preparedness and Remediation

Water damage to library resources can occur in many ways. Water can come directly from torrential rains, flooding, leaks, or failed hydraulic and conditioning systems, or indirectly from extinguishing a fire [126]. In all cases, contact with water causes absorption, adhesion, curling, and swelling of paper, migration of inks and dyes, and microbiological contamination [127]. Damp paper is very susceptible to mold, which begins to grow within 24–28 h, especially if temperatures are warm. Once established, mold contamination is difficult to remove.

Climate change has led to a global increase in the frequency of forest fires [128], floods, hurricanes, and storms of all kinds [129]. It is hoped that librarians will become more knowledgeable about disaster preparedness so as to minimize the problems that fungi can pose to library buildings and their contents. Flooding incidents in libraries frequently occur from slow leaking pipes, water penetration through cracks in walls, and weather incidents associated with storms. In ordinary times, buildings and collections are most at risk during renovations and moves. Many molds are fast growing, so when water damage occurs, it is important to act as soon as possible. Dehumidifiers can reduce the moisture content of the air in wet areas. Practical handbooks for developing disaster management plans include Barton and Wellheiser [130], Morris [131], and Alire [132]. Table 3 provides various remediation approaches used to treat water-damaged library books and their cost estimates and time requirements.

Control methods for biodeterioration are generally categorized into physical, mechanical, and biochemical approaches. Mechanical treatments typically involve removing or displacing biodeteriogens through manual techniques or physical tools such as vacuum cleaners, scalpels, and spatulas. However, these methods have often been ineffective in fully preventing microbial regrowth. Alternatively, various physical techniques—including low-frequency electrical systems, heat, and ultraviolet (UV) radiation—have been employed to preserve cultural heritage sites [133]. While UV radiation is used to inhibit fungal growth, gamma radiation has been applied to sterilize microbial populations. Nevertheless, the sterilizing effects of gamma radiation are temporary, and UV radiation poses safety risks to users [134].

Various compounds such as aldehydes, phenols, amides, acids, alcohols, and quaternary ammonium compounds are commonly used as biocides. Effective biocides function by penetrating microbial cells and inhibiting or killing the organisms internally. Some chemicals act as permeation enhancers, increasing the permeability of cell membranes to facilitate biocide entry [135]. Ethanol vapor, for example, has proven effective in disinfecting paper-based materials. Gilot et al. (2012) showed that exposing books and brochures to 70% ethanol vapor in a closed environment successfully suppressed fungal growth without compromising paper quality [136]. However, it is important to note that only a few biocides offer long-lasting protection, and some may lead to microbial resistance and subsequent recolonization. Additionally, certain biocides can negatively affect the integrity of treated materials [134].

It is essential to consider that the use of biocides is strictly regulated and varies significantly between countries and regions. In the European Union, for example, only biocidal products that have been assessed and approved under the EU Biocidal Products Regulation (BPR, Regulation (EU) 528/2012) can be legally used. These approvals are managed by the European Chemicals Agency (ECHA), which maintains a searchable database of authorized substances and products. This means that conservators and scientists must consult national or regional regulatory lists to ensure compliance before selecting a treatment [137]. In the context of paper conservation, common active substances such as quaternary ammonium compounds, isothiazolinones, or alcohol-based disinfectants may be permitted for use, but their application must adhere to concentration limits, safety protocols, and material compatibility guidelines [138]. Some products previously used in conservation, such as pentachlorophenol or certain organotin compounds, are now banned or heavily restricted worldwide due to toxicity concerns and environmental impact [139]. Therefore, integrating regulatory awareness into conservation planning is not only a matter of legal compliance but also a best practice for health and heritage safety [137].

Before the advent of widespread air conditioning, fumigation was a mainstay for preventing mold growth in libraries [140]. For example, vaporized phenyl mercuric acid [141] and *o*-phenylphenol (OPP) [142] were both evaluated as fungal growth retardants. Vaporized formaldehyde was employed after the devastating blaze at the Union of Soviet Socialist Republics (U.S.S.R.) Academy of Sciences Library in Leningrad in 1988 [143]. Ethylene oxide has also been used despite concerns about its activity as a known carcinogen in experimental animals [144,145]. An early published report on fungal contamination of libraries from 1932 concerned the Los Angeles Public Library. Thousands of bound volumes of newspapers developed serious “mildew” problems during the non-airconditioned summer months. At that time, fumigants were recommended for curbing fungal contamination. Nevertheless, the librarian writing about the problem recognized their drawbacks, and close to a century later, her words are still relevant: “… of the many fumigants available few could be used because of the number of volumes affected, and there are very few fumigants that have no disagreeable odor, are innocuous to human life, or health, or the bindings or paper stock” [17]. Contemporary efforts are underway to develop packaging boxes for storing cultural heritage materials that contain antimicrobial materials that are safer than traditional fumigants to prevent the growth and reproduction of fungal colonies [146].

Currently, freezing is considered the best way to stabilize water-soaked paper against further deterioration, thereby providing valuable time in which decisions can be made as to whether to dry and restore or discard damaged materials. Freezing renders mold spores and vegetative cells dormant, stabilizes soluble inks and dyes, and prevents coated paper sheets from sticking [147,148]. The choice of the subsequent treatments for water-damaged library items depends on different factors, including but not limited to the level and cause of damage, the rarity/scarcity of the damaged items, the number of volumes and documents impacted, and the extent of the available budget, personnel, and drying services. When deciding on the appropriate recovery method, it is also important to consider the recommendations of a preservation administrator or conservator experienced in disaster recovery [149].

Before starting the treatment, possible contamination with dirt, rust, or salinity should be assessed so that materials can be rinsed before freezing to remove impurities. Then, most library resources can be dried either through sublimation or evaporation. Liquid water will evaporate. Under appropriate freeze-drying conditions, water in a solid state (i.e., ice) will sublimate [150]. Freeze drying does not further damage the treated items and yields dried products in good condition while limiting biodeterioration [127]. For successful freeze drying, the wet materials must be placed in a freezer capable of rapid freezing as soon as possible. To reduce distortion and facilitate drying, the temperature in the freezer should be −40 to −23 °C. Freeze drying can only be performed for a small number of materials at a time. Moreover, the drying stage can take several months depending on the temperature of the freezer and the degree of water damage [149].

Freeze drying also has potential pitfalls, the most common of which is embrittlement due to the over-drying of items [150], and freeze drying is obviously not suitable for non-book resources such as computers, video and audio tapes, discs, and CDs, which must be air dried [151]. While freeze drying is widely used to stabilize water-damaged paper substrates, it is important to note that this method does not serve as a definitive means of decontamination, as it fails to inactivate fungal cells or their extracellular enzymes. Preserving fungal viability alone is insufficient to ensure long-term material stability; assessing the retention of enzymatic functionality is equally crucial. Sundari and Adholeya (2000) [152] demonstrated that key extracellular enzymes, such as cellulases, remained active in several fungal species following freeze drying. Their qualitative assays showed that lyophilized mycelia exhibited enzymatic activity comparable to that of non-lyophilized controls [152]. Similarly, Grzegorczyk et al. (2018) found that freeze drying preserved the viability of Trichoderma strains and, notably, that hydrolytic enzymes such as cellulase and xylanase remained stable or even displayed increased activity after freeze drying and storage [153]. These findings highlight a significant limitation of freeze drying; the persistent enzymatic activity of fungi can continue to degrade cellulosic materials, even in the absence of visible fungal growth.

On the other hand, vacuum freeze drying is convenient for large numbers of wet books and materials with coated papers and water-sensitive inks. The recovery plan for water-soaked materials by vacuum freeze drying starts with packing them in waterproof bags that are then placed in boxes and kept frozen until they are processed and transported to the restoration facility. Once the samples are ready for processing, they are first thawed and washed. Washing is performed page-by-page with gentle brushing. To allow excess water to drain, materials are spread on a table before being placed on a drying cart. The washed materials are then refrozen and moved to a vacuum freeze-drying chamber, which uses vacuum pumps to remove the air inside until the air pressure drops below 533.28 Pa while keeping the temperature below 0 °C [154]. Sublimation dries by turning ice into vapor, thereby bypassing the liquid phase and preventing further damage to the material. As this technique requires the use of sophisticated equipment, it is more expensive than other methods and is generally reserved for rare and unique wet materials [149]. Vacuum freeze drying utilizes a vacuum thermal drying chamber and is suitable for many materials while also being cost-effective [149,155].

Air drying is a straightforward process by which materials are simply spread out and dried under normal indoor environmental conditions of 40–55% relative humidity and temperatures of 20–25 °C. This approach is suitable for small amounts of moderately damaged materials [149]. Fans should be run continuously to maintain air recirculation, and pages should be interleaved with absorbent paper to accelerate the drying process [156]. Nonetheless, a key problem in air drying is that it is very laborious, with an extensive need for verification, interleaving, re-verification, and re-interleaving and often results in distorted bindings and blocks of text [150]. Although this method yields the least favorable results, it does not require sophisticated equipment and is the least expensive [149]. Freeze drying is the preferred way to recover paper-based materials.

When water damage is limited, another low-cost approach is desiccant dehumidification, in which materials are dried while still on their shelves using large dehumidifiers while the temperature and relative humidity are constantly controlled. During dehumidification, dry air is introduced into the book storage area while moisture is removed. Unlike normal dehumidifiers, desiccant dehumidifiers force very dry air into specific spaces and pump out moist air to speed up the drying process. Books can be dried on shelves without the need to relocate them, but this treatment is not practical for most collections because soluble materials will bleed, and coated materials will clog [150].

Once damaged collections are processed, subsequent rehabilitation procedures include binding, applying new labels, boxing the documents, removing dirt and debris, refolding, and eventually reshelving. Ideally, the treated materials should be placed in an open, well-ventilated, and air-conditioned space and separated from other materials [149]. This “rehabilitation area” allows the condition of the books to be assessed and then sorted according to their readiness to return to their normal housing environment. The rehabilitation area should also be maintained under adequate indoor environmental conditions (temperature below 18 °C and relative humidity of 30 to 40%). Throughout the process, regular inspections for mold contamination should be carried out by qualified personnel [124,154]. When regular inspections reveal mold contamination in library collections, immediate and appropriate actions are necessary to prevent further deterioration and contamination of unaffected materials. The first step involves isolating the affected materials in a designated area to prevent cross-contamination [157]. The extent of contamination should then be assessed, determining whether individual items can be salvaged through remediation or if severe damage warrants disposal [158]. Once isolated, environmental stabilization is critical to inhibit further mold growth [159]. After stabilization, materials should be cleaned using appropriate methods and employing specialized techniques for delicate or heavily affected items [157]. In cases where cleaning poses risks to the integrity of materials, professional conservation services may be required [159]. For items beyond restoration, proper disposal protocols must be followed to ensure that mold spores do not spread to unaffected collections.

Illustrative examples of libraries that have experienced water disasters are described below, followed by brief descriptions of different remediation processes. One of the first published reports about freeze drying as a successful remediation process for water-damaged library holdings followed rescue operations after a fire at the Greenland Regional Library in Godthab in 1968. The fire was rapidly extinguished, but with accompanying extensive water damage to books, handwritten manuscripts, and letters. Fortuitously, the cold winter temperatures in Greenland froze the water-soaked library holdings. The icy materials were transported to Copenhagen and kept frozen for two more years. Over this time, printed books were gradually air dried, while more valuable items, such as manuscripts, handwritten letters, and photographs, were freeze dried to prevent ink from running during the drying process. It was later reported that “The drying process gave perfect results for all the written documents” but not the photographs [160].

Another disaster recovery case concerned The College of Physicians in Philadelphia, Pennsylvania, in 1978, when a combination of heavy rain and a faulty roof damaged a collection of more than 4000 rare books. Approximately 99% of the collection was saved via vacuum freeze drying. Before drying, the books were wrapped in plastic bags and frozen at −12–22 °C. The books were then transported to storage at −45 °C in a freezer plant to halt further deterioration [161]. Similarly, in 1995, 250 rare books at the University of Maryland became water-soaked. The books were initially frozen and then vacuum freeze dried. Unfortunately, some of the older volumes ended up with smudges and brittle leaves. To remove the stains and return the leaves to their flexible state, the books were submerged in recalcified water buffered with magnesium bicarbonate. The books were subsequently drained, pressed by hand, and delicately reshaped. Groups of pages were carefully opened, and an interleaving polypropylene and cellulose fiber material was inserted. The books were finally air dried, and the reported results were excellent [147].

The greatest water-related library disaster in US history occurred at Colorado State University’s (CSU) Morgan Library in Fort Collins, Colorado. The library was flooded on 28 July 1997 because of a series of summer thunderstorms. CSU’s water-damaged books were transported frozen via overland trucking from a commercial cold storage facility in Wyoming to another commercial cold storage service in Fort Worth, Texas. The books were thawed, washed in clean running water to remove mildew and dirt, pressed to remove excess water, and then refrozen. Three freeze-drying chambers were employed concurrently. The frozen books were carried inside the chambers and heated to around 35 °C. By keeping the pressure within each chamber below 533.28 Pa and the temperature between 21 and 26 °C, much of the frozen water sublimed directly into vapor. Following the drying process, the books then were sent to a sterilizing facility for gamma radiation treatment. The impacts of mold on the collection worsened with each day that the books stayed damp in the library, with visible spore germination increasingly evident on book bindings and text borders. The resulting discoloration spread to the leaves of the texts until the books were frozen and the mold went dormant. Books recovered during the first few days of the pack-out had little or no text staining, but books retrieved in the last few days of the recovery tended to have twenty or more pages of damage [162].

In another example from the summer of 2012, the art and architecture library at Kansas University was flooded because of extreme drought conditions that, somewhat paradoxically, caused a main water break. Subsequently, more than 17,000 water-damaged volumes were vacuum freeze dried. The following summer, 4500 additional volumes were soaked because of a mechanical failure in the science and social science library, necessitating another round of treatment, while also demonstrating that large-scale recovery of library collections, particularly books, was possible [163].

When remediation efforts are underway, human safety is the top priority. To prevent the inhalation of mold spores and other aerial contaminants, masks should be provided. Workers should use gloves when handling wet material. Throughout the remediation process, library workers and patrons should be informed about how they will be impacted. Some remediation plans may require the relocation of library occupants. The U.S. Environmental Protection Agency has prepared several useful manuals and fact sheets that describe methods for safe cleaning of mold contamination [164]. Librarians and archivists have formed many excellent organizations that provide information about the preservation of library materials. The Council on Library and Information Resources (CLIR) grew out of a merger between the earlier Council on Library Resources and the Commission on Preservation and Access. A parallel organization in Europe is the European Commission on Preservation and Access (ECPA). The global organization representing libraries is The International Federation of Library Associations and Institutions (IFLA), which is headquartered in The Hague in The Netherlands. Each of these organizations hosts a helpful website. Organizations that focus specifically on conservation and restoration include the European Preservation Information Center, the Internal Council of Archives, the International Federation of Library Associations and Institutions (IFLA), and the “Memory of the World” Program of UNESCO. In the United States, The Library of Congress has a center for Preservation and Conservation as does the IFLA. Mary Ann Florian is a conservator who has written several useful books that deal with what she calls “The Heritage Eaters” [165,166]. A useful infographic on mold and cultural heritage materials is available at: https://ccaha.org/resources/mold-and-cultural-collections (accessed on 30 April 2025).

Moreover, The International Organization for Standardization (ISO) offers guidelines on the preservation of library holdings and appropriate environmental conditions for the library space. For instance, ISO/TR 19814:2017 [167] provides instructions and recommendations for the implementation, maintenance, and improvement of library and archive collection preservation through procedures for managing collections in the conservation facilities, reading rooms, stacks, on exhibit, and during transportation. The report also includes guidance for suitable enclosures and containers for the library’s small and large volumes. It also focuses on effective library resource management to reduce several hazards, including catastrophic loss due to flooding or fire [168]. Likewise, ISO/TR 19815:2018 [169] sets out a framework for determining the appropriate environmental conditions for library collections, considering the specific climatic zones in which they are housed. Since there are no distinct conditions that are appropriate for all collections in all circumstances, environmental specifications are customized to meet the unique needs of each collection, the available resources of the institution, its operational context, and the local climate [170]. In addition, national and international libraries and archival organizations that host useful websites for the conservation of library materials are listed in the Supplementary Material (Table S1).

Digitization is a crucial recovery strategy for newspapers, magazines, and books that have suffered water damage in libraries. When physical materials are too fragile for conservation, scanning and digital preservation ensure continued access to their content [171]. Newspapers and magazines, often printed on acidic paper, are particularly vulnerable to water exposure, leading to rapid deterioration. By converting them into high-resolution digital files, institutions can mitigate the risk of total information loss [172]. For books with moderate damage, digitization can complement conservation efforts by reducing the need for physical handling. Optical Character Recognition (OCR) technology further enhances accessibility by enabling text searches within scanned documents [173]. Additionally, digital backups provide resilience against future disasters, allowing libraries to maintain their collections in a format less susceptible to environmental threats [174]. However, successful digitization requires proper drying and cleaning of materials before scanning, as mold growth and structural damage can compromise image quality. Libraries must also invest in long-term digital preservation strategies, including secure storage, metadata creation, and periodic file migration to prevent data loss [172]. While digitization cannot replace the original artifacts, it ensures that valuable historical and cultural records remain accessible for future generations.

**Table 3:** Cost estimates and time requirements for remediation approaches to water-damaged library materials.

Remediation Approaches	Estimated Cost	Estimate of the Time Required	References
Biocides	Low to moderate	Minutes to hours, depending on the product used	[134,136]
Fumigation (historical method using chemicals like formaldehyde or ethylene oxide)	High	Immediate effect, but safety concerns limit use	[141,144]
Dehumidification (using large desiccant dehumidifiers)	Low to moderate	Hours to days, depending on moisture levels	[150]
Freezing (stabilizing wet materials against further deterioration)	Moderate	Immediate freezing; materials can remain frozen indefinitely before further processing	[147,148]
Air drying (spreading materials in a controlled indoor environment)	Low (but labor-intensive)	Several days to weeks	[149,150]
Freeze drying (drying materials by sublimation)	Moderate to high	Weeks to months	[127,147,148,150]
Vacuum freeze drying (more efficient large-scale sublimation drying)	High	Weeks to months	[149,154,155]
Gamma radiation (used for sterilizing mold-affected books)	High	Immediate effect, requires specialized facilities	[159]

## 6. Threats to Human Health

Most fungi cannot grow at the human body temperature. Therefore, of the estimated millions of fungal species believed to exist, only a few hundred are ever associated with human disease (mycosis) [175]. Moreover, the human innate immune system is good at warding off even these species, so people with healthy immune systems do not succumb to fungal pathogens. Unfortunately, for people with weak and failing immune systems, several species of fungi common to libraries are potentially pathogenic, including several species of *Aspergillus* [176]. While fungal infections are rare, unfortunately, fungal allergies are common [177]. Moreover, exposure to fungal spores can exacerbate asthma [171]. Meta-analyses have shown that respiratory exposure to *Aspergillus*, *Cladosporium*, and *Penicillium* species, all of which are common in indoor environments, poses an increased risk for allergies and asthma in susceptible populations [178,179].

Finally, some fungi produce poisonous metabolites called mycotoxins [96,180]. While most of the human risk of mycotoxin exposure comes through foods, a few of these toxins are also associated with airborne spores [180,181]. Respiratory exposure to mycotoxins may be linked to an array of ill-defined negative health responses often called “sick building syndrome” or “building-related illness” [3,182,183,184].

The possible potential health impacts of exposure to indoor fungi are beyond the purview of this primer. Nevertheless, common sense dictates that librarians with allergies, asthma, and compromised immune systems should avoid contact with the high concentrations of fungal spores that are associated with water events and that librarians and patrons alike should wear masks when there is a danger of breathing in unusually high levels of airborne fungal propagules. Similarly, microbiologists called in as experts to help with water problems in libraries should exert caution while they sample and otherwise help with the clean-up efforts.

On the other hand, a number of conventional biocides used in the conservation of cultural heritage materials are associated with notable health hazards. Quaternary ammonium compounds, for instance, can irritate the skin, eyes, and mucous membranes when used at concentrations exceeding 6%. Azole-based substances have been found to pose ecological risks, particularly to aquatic life, which in turn raises environmental and public health concerns. Similarly, phenolic compounds may lead to both acute and long-term health effects, while heavy metals such as copper and mercury are recognized for their high toxicity to humans. These concerns have spurred growing interest in natural biocides as potentially safer alternatives [185]. Nonetheless, the safe use of any biocidal product demands thorough risk evaluation, as insufficiently assessed applications may present serious health risks [186].

## 7. Conclusions

Millennia ago, Vitruvius, a famous Roman architect, wrote: “The rooms and libraries should be facing the sunrise, for their use demands morning light, and furthermore so that the books do not decay as much in these libraries as those that face the noon sun. The same wind-borne humidity that gives birth to and nourishes worms also causes books to mold” [1]. Many legitimate reasons exist for concerns about indoor mold contamination, the primary of which are the potential for negative health impacts on people using and working in libraries and the biodeterioration of library holdings. When mold growth threatens the contents of a library and the health of the people who use the library, the information summarized in this primer can serve as a resource for microbiologists, environmental scientists, and mycologists who are brought in to help.

An Arabic saying contends that “A book is like a garden carried in the pocket”. Like gardens which regularly suffer from a variety of blights, rusts, smuts, spots, molds, and mildews, libraries are subject to microbial assaults. When serious water damage occurs due to floods, plumbing failures, sprinkler malfunction, storm damage, or the aftermath of the use of water hoses to extinguish a fire, immediate and aggressive remediation is essential. People with allergies, asthma, or compromised immune systems should avoid contact with moldy materials in libraries and elsewhere. It is impossible to make a library entirely mold-proof, but awareness of protective measures should be part of building planning and maintenance [187]. Librarians and mycologists agree that libraries, like gardens, require constant tending.

Water is essential for fungal proliferation, so keeping libraries dry is the single most important measure for preventing mold contamination. In the future, architects and other professionals who build libraries should be more cognizant of the importance of minimizing potential water damage. In humid climates, HVAC systems should be designed to reduce condensation as well as ensure improved air filtration and adequate outside air ventilation rates. Furthermore, building systems and enclosure assemblies should have accessible concealed spaces so that hidden colonies of mold can be accessed.

Good building maintenance is also fundamental. Roof and plumbing leaks should be remedied immediately. With respect to major storms, flooding, and water damage associated with extinguishing fires, wet books and other materials should be immediately frozen, providing time to create salvage plans for damaged items. When mold is discovered, environmental scientists with expertise in indoor mold problems should be consulted. A combination of molecular and morphological identification techniques should be employed for accurate species identification so as to evaluate if molds are a danger for toxin and allergen production. In the United States, several commercial companies specialize in mold testing and remediation. Many universities and government laboratories employ microbiologists who usually are willing to help address mold problems. We hope that this article will serve as a primer for scientists who are asked to help librarians with mold contamination.

## Data Availability

No new data were created or analyzed in this study. Data sharing is not applicable to this article.

## Supplementary Materials

The following supporting information can be downloaded at: https://www.mdpi.com/article/10.3390/biology14080911/s1, Table S1: Organizations with Useful Websites for the Conservation of Library Materials.
