# Supplementary material for: Drenched Pages: A Primer on Wet Books
Source: Biology (Basel). 2025 Jul 22;14(8):911. doi: 10.3390/biology14080911 (PMC12383548; doi:10.3390/biology14080911)
Supplement: Supplementary file 1 [file biology-14-00911-s001.zip › biology-3689865-supplementary.pdf]

Supplementary material

Table S1. Organizations with Useful Websites for the Conservation of Library Materials

**Library**	**Location**	**Website**
Austrian National Library	Vienna, Austria	https://www.onb.ac.at/en/more/institute-for-conservation/conservation/books
British Library	London, UK	https://www.bl.uk/conservation
Harvard Library	Massachusetts, USA	https://preservation.library.harvard.edu/preservation-libraries
Herzog August Library	Wolfenbüttel, Germany	https://www.hab.de/en/conserving-holdings/
Library And Archives, Canada	Ottawa, Canada	https://library-archives.canada.ca/eng/corporate/about-us/building-projects/new-preservation-facility/Pages/preservation-centre.aspx
Library of Congress	Washington DC., USA	https://www.loc.gov/preservation/emergprep/dry.html
Library of Parliament	Ottawa, Canada	https://lop.parl.ca/sites/PublicWebsite/default/en_CA/About/Spotlight/RareBooks
Melk Monastery Library	Melk, Austria	https://www.thebestcolleges.org/amazing-libraries/
National Archives	Washington DC., USA	https://www.archives.gov/preservation
National Diet Library	Tokyo, Japan	https://www.ndl.go.jp/en/preservation/collectioncare/scheme.html
New York Public Library	New York, USA	https://www.nypl.org/collections/preservation-division/conservation-treatment#:~:text=Conservators%20in%20the%20Barbara%20Goldsmith,for%20current%20and%20future%20generations.

State Library of Victoria	Melbourne, Australia	https://www.slv.vic.gov.au/search-discover/conservation-guides
The Bodleian Library	Oxford, UK	https://www.bodleian.ox.ac.uk/about/libraries/our-work/conservation
The Morgan Library Museum	New York, USA	https://www.themorgan.org/thaw-conservation-center
Thomas Fisher Rare Book Library	Toronto, Canada	https://fisher.library.utoronto.ca/services/conservation
Trinity College Library	Dublin, Ireland	https://www.tcd.ie/library/preservation/
Yale University Library	Connecticut, USA	https://guides.library.yale.edu/preservation
